# l‑Cysteine-Glutathione Mixed Disulfide, a Novel Bioavailable Sulfhydryl-Modified Glutathione Precursor, Protects against Early Liver Injury Induced by Short-Term Hypercholesterolemia

**DOI:** 10.1021/acs.chemrestox.5c00272

**Published:** 2025-10-23

**Authors:** Laura Martínez-Gili, Raquel Fucho, Francisco Caballero, Susana Núñez, Hala Saeed Jaara, Cristina Alarcón-Vila, Naira Rico, Herbert T. Nagasawa, Carmen García-Ruiz, José C. Fernández-Checa, Sandra Torres

**Affiliations:** † Department of Molecular and Cellular Biomedicine, Institute of Biomedical Research of Barcelona (IIBB), 54448 Spanish National Research Council (CSIC), Barcelona 08036, Spain; ‡ Liver Unit, Hospital Clinic I Provincial de Barcelona, Institut d’Investigacions Biomèdiques August Pi i Sunyer (IDIBAPS), Barcelona 08036, Spain; § Center for the Study of Liver and Gastrointestinal Diseases (CIBERehd), Carlos III National Institute of Health, Madrid 28029, Spain; ∥ CORE Laboratory, Biochemistry and Molecular Genetics Department, Biomedical Diagnostic Centre, Hospital Clínic, Barcelona 08036, Spain; ⊥ Center for Drug Design, University of Minnesota, Minneapolis, Minnesota 55455, United States; # Division of Gastrointestinal and Liver Diseases, Department of Medicine, Keck School of Medicine, University of Southern California, Los Angeles, California 90033, United States

## Abstract

Oxidative stress contributes to the damage of biological molecules and is linked to the development of multiple diseases, including liver disorders, such as metabolic dysfunction-associated steatotic liver disease (MASLD). In mammals, reduced glutathione (GSH) is a pivotal antioxidant that regulates cellular responses to redox imbalances caused by reactive oxygen and nitrogen species. The presence of reduced GSH within mitochondria is especially crucial for preserving the organelle’s routine performance by eliminating hydrogen peroxide generated under both physiological and pathological conditions. Cumulative evidence indicates that MASLD is associated with a diminished mitochondrial GSH (mGSH) pool, attributed to alterations in mitochondrial membrane fluidity due to cholesterol accumulation. Therefore, strategies aimed at boosting mGSH may offer therapeutic benefits against MASLD-associated liver injury. This study aims to investigate whether l-cysteine-glutathione disulfide (l-CySSG), a proposed GSH donor and precursor, can effectively restore total and mGSH in vitro and in vivo in mice fed cholesterol-enriched (HC) or methionine-choline-deficient (MCD) diets. Additionally, *S*-adenosylmethionine (SAM), a compound that serves as both a GSH precursor and a membrane fluidizer, along with *N*-acetylcysteine (NAC), a GSH precursor by providing cysteine, was used as the control molecules in the study. Our findings show that l-CySSG has great potential as a liver protector, especially due to its good oral bioavailability. Although it does not restore GSH levels in the mitochondria as efficiently as SAM does, l-CySSG can still offer protection against liver damage, possibly through mechanisms that are not yet fully understood. Overall, l-CySSG emerges as a promising alternative for treating conditions related to oxidative stress and mitochondrial dysfunction, paving the way for future research and therapeutic development.

## Graphical abstract

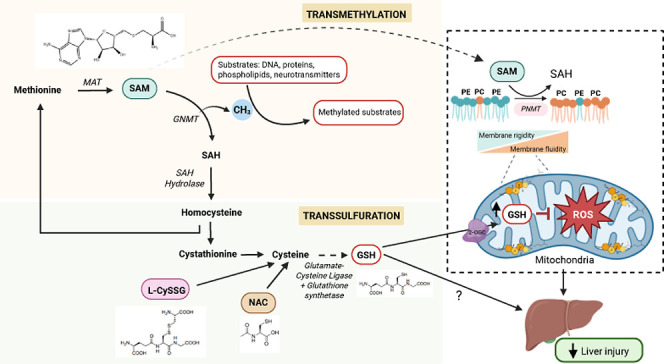

## Introduction

Glutathione (GSH) is a crucial molecule in cellular physiology and biochemistry, recognized as the most prevalent nonprotein thiol within cells. It is composed of three amino acids: glutamate, cysteine, and glycine. The functional capabilities of GSH are primarily attributed to the sulfhydryl group of cysteine, which plays a significant role in various processes, such as detoxification of electrophiles, maintenance of redox balance, and reduction of protein thiol oxidation. Meanwhile, glutamate and glycine serve to safeguard the molecule against intracellular degradation.^1^

The synthesis of GSH occurs in the cytosol through two sequential enzymatic reactions that require ATP hydrolysis. The first step involves the combination of l-glutamate and l-cysteine, catalyzed by the enzyme glutamate-cysteine ligase (GCL), followed by the addition of l-glycine, facilitated by GSH synthetase (GS).^2^ The rate-limiting step is the initial enzymatic reaction, which is regulated by the availability of cysteine and the concentration of GSH itself, as excess GSH can inhibit GCL through a nonallosteric mechanism.^3,4^ Notably, the liver possesses a unique capability to convert methionine to cysteine via the transsulfuration pathway, enabling it to compensate for cysteine deficiency.^5–7^

Once synthesized in the cytosol, GSH is subsequently distributed to various cellular organelles. It is particularly vital for preserving the functionality of mitochondria, which are the primary source of ROS resulting from oxygen consumption in the respiratory chain.^8^ Under normal circumstances, the antioxidant defense system of mitochondria can effectively mitigate these species; however, failure to do so may lead to oxidative stress, cell death, and organ damage. The lack of the enzyme catalase within this organelle in most tissues, including the liver, renders the presence of mitochondrial GSH (mGSH) essential, as a main mechanism for reducing hydrogen peroxide in mitochondria involving the GSH redox cycle (GSH peroxidase and GSH reductase), which utilizes GSH as a cofactor.^9^ Mitochondria do not synthesize GSH de novo and mGSH derives from the activity of solute carriers that transport GSH from cytosol to mitochondrial matrix, which is sensitive to changes in membrane fluidity, physiologically determined by the presence of cholesterol in the membrane bilayer.^9^ Various candidates have been suggested to perform this task such as SLC25A11, whose function is directly regulated by membrane fluidity.^10–12^ Unlike SLC25A11, the activity of SLC25A39, another putative mGSH carrier, has not been documented to depend on appropriate mitochondrial membrane fluidity nor its kinetics characteristics and substrate specificity have been reported.^13–16^

Research has shown that conditions like metabolic dysfunction-associated steatotic liver disease (MASLD) and diseases linked to hypercholesterolemia are characterized by a selective reduction in mGSH levels, often due to changes in mitochondrial membrane fluidity.^17–19^ When mGSH is depleted, mitochondria become more vulnerable to excessive ROS production, which can trigger oxidative stress and potentially lead to inflammation, fibrosis, and cell death. Restoring mGSH levels, therefore, may offer a promising therapeutic strategy to mitigate the symptoms and progression of these disorders.

In this study, we used two dietary MASLD models: the methionine-choline deficient diet (MCD), which is a widely recognized nutritional model for MASH, despite causing weight loss, and the hypercholesterolemic (HC) diet, which mimics conditions related to hyperlipidemia and hypercholesterolemia, both of which are risk factors for cardiovascular and liver diseases, such as fatty liver disease and atherosclerosis. These dietary interventions lead to a reduction in mitochondrial membrane fluidity through distinct mechanisms, thereby hindering the transport of GSH into the mitochondria.^20–22^ Specifically, in the context of the MCD diet, both methionine and choline participate in synthesis pathways of phosphatidylcholine (PC), a principal lipid component of cellular membranes, whose presence is crucial for the formation of very low-density lipoproteins. Methionine serves as a precursor for *S*-adenosylmethionine (SAM), which is essential for the methylation of phosphatidylethanolamine (PE) to generate PC. Additionally, choline combines with glycerophosphate and fatty acids to form PC. Consequently, a deficiency in methionine and choline results in mGSH depletion by reducing the PC/PE ratio in membranes, thereby affecting their fluidity, as previously reported.^21,22^ It has been demonstrated that in the context of a HC, an elevation of free cholesterol within mitochondrial membranes disrupts their fluidity, hinders the transport of GSH into the mitochondria, and disrupts the assembly of mitochondrial supercomplexes, leading to impaired oxidative phosphorylation.^20^

Our study is designed to assess whether l-CySSG protects against MCD and HC-mediated liver injury and homeostasis and compartmentalization of GSH. We find that l-CySSG, a metabolic byproduct found in mammalian cells^23,24^ (Figure 1), protects against liver injury in both models and this protective effect exhibit similarities and differences compared to SAM and NAC in relationship with the replenishment of mGSH.

**Figure 1:**
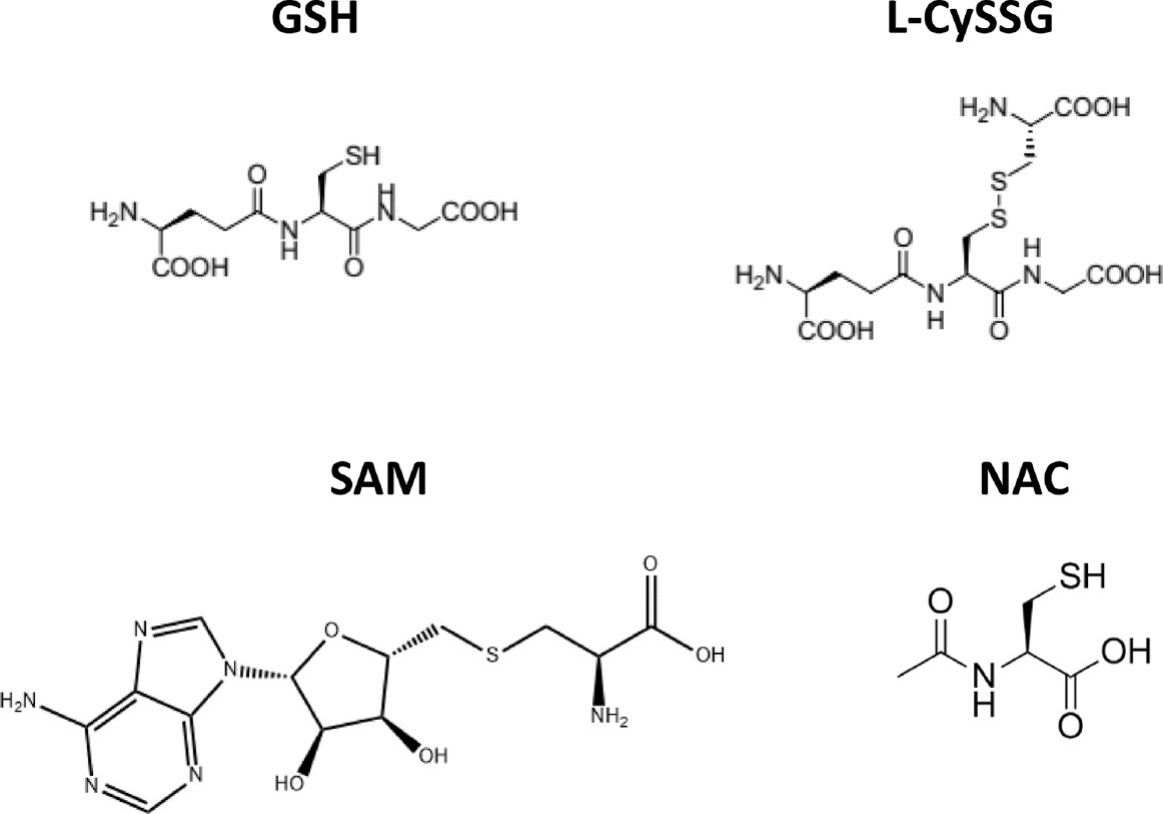
GSH prodrugs.

## Materials and Methods

### Animal Models and Treatments

Wild-type C57BL/6J male mice (8–10 weeks old) were obtained from Charles River Laboratories (Wilmington, MA). Mice were fed for 2 days with either chow (control diet) or HC, containing 2% purified cholesterol with 0.5% sodium cholate supplementation, and with MCD diet (Dyets Inc.) from 2 to 15 days. Animals that were maintained on a chow diet served as the control group. On the third day of the HC diet, at 09:00 AM, the first dose of l-CySSG (1.25 mmol/kg, administered i.p.) was given to the animals. One hour later, lipopolysaccharide (LPS) was introduced to induce liver injury at a dosage of 5 mg/kg, also via i.p. injection. Subsequently, 30 min after the LPS administration, a second dose of the l-CySSG prodrug was administered i.p. at a concentration of 2.5 mmol/kg. For those treated with LPS, the animals were sacrificed 24 h postinjection. In the cases involving SAM and NAC treatments, LPS (5 mg/kg) was injected i.p. on the third day of feeding at 09:00 AM. Three hours following the LPS injection, either SAM (15 mg/kg) or NAC (2.5 mmol/kg) was administered i.p. Animals were sacrificed 24 h after the LPS injection for subsequent liver and blood analyses. Animals were housed in temperature- and humidity-controlled rooms and kept on a 12 h light/dark cycle. All animal experiments were performed following the protocols evaluated and approved in accordance with the Animal Care Committee of the Hospital Clinic-Universidad de Barcelona and conformed to the highest international standards of humane care of animals in biomedical research (Ethics Approval Number: CEEA 405/13, 7133; CEEA 204/24).

### Hepatocyte Isolation and Treatment

Hepatocytes from MCD-fed mice for 2 days were isolated by collagenase perfusion with a flow rate of 7–9 mL/min and cultured at a density of 2 × 10^6^ cells on 60 mm dishes coated with rat tail collagen. Hepatocytes were cultured in DMEM/F12 culture media (Gibco, 21331-020) supplemented with 10% FBS for the first 3 h post isolation, together with 10,000 U/ml penicillin–streptomycin (Gibco, 15140-122), 200 mM l-glutamine (Gibco, 25030-024), 7.5 mM d-glucose (Sigma, G6152), 150 mM Hepes pH 7.4 (Sigma), and 1 mM methionine (Sigma) (except for MCD hepatocytes) that were used as culture medium. In some instances, hepatocytes from control mice were treated with 30 μM Cholesterol water-soluble (Sigma, C4951) and 10 nM Acat inhibitor (Santa Cruz, sc-203892) for 16 h before antioxidants treatments. This in vitro approach mimics cholesterol accumulation in vivo with the HC diet. Hepatocytes were exposed to NAC, SAM, or l-CySSG (10 mM, pH adjusted to 7.4 with 10 N NaOH) and were collected at two different time points: 3 and 6 h. Hepatocytes were fractionated into cytosol and mitochondria by digitonin permeabilization, as described previously.^25^

### Mitochondria Preparation and GSH Quantification

Highly purified mitochondria from the liver homogenate were prepared by rapid centrifugation through a Percoll density gradient. Briefly, mice were anesthetized with sodium pentobarbital (60 mg/kg), and the livers were dissected. All the following steps in mitochondrial isolation were performed at 4 °C. The dissected tissue (1 g) was minced into 9 mL of ice-cold isolation medium (Buffer A) containing 225 mM mannitol, 75 mM sucrose, 0.1 mM EGTA, 1 mg/mL FFA-BSA, and 10 mM HEPES, pH 7.4. Next, the minced tissue was processed using a drill-driven Teflon Dounce homogenizer at 1200 rpm (4 times up and down). The resulting homogenate was centrifuged for 15 min at 700 *g*. The supernatant was then centrifuged at 10,000*g* for 15 min. The pellet was resuspended in 0.5 mL of the medium (Buffer B) containing 395 mM sucrose, 0.1 mM EGTA, and 10 mM HEPES, pH 7.4. This fraction contains mitochondria together with the presence of other organelles (crude mitochondria). The homogenate was layered in an ultracentrifuge tube over preformed discontinuous Percoll gradients consisting of a bottom layer of 3 mL of 60% Percoll in buffer B and a top layer of 7 mL of 26% Percoll in buffer B. The gradients were spun at 15,500 rpm for 35 min in a Beckman SW-41 rotor. The mitochondrial fraction, appearing at the interface between the bottom two layers, was gently collected with a glass Pasteur pipet, and resuspended in a fresh tube in 20 mL of buffer B, centrifuged for 15 min at 10000*g*, and recentrifuged under the same conditions. The supernatant was removed, and the pellet containing mitochondria was resuspended in 350 μL of buffer A. This fraction contains pure mitochondria. Mitochondrial protein concentration was determined using a Pierce BCA kit. Total and mitochondrial GSH were quantified using Ellman’s reagent as described before.^26^ Briefly, liver homogenate and mitochondria samples were diluted in 10% trichloroacetic acid and then centrifuged. The concentration of total GSH (reduced and oxidized) was determined spectrophotometrically at 412 nm by kinetic enzymatic analysis with GSH reductase. GSH levels were normalized by protein content determined by the Bradford protein assay.

### Biochemical Determinations

Alanine aminotransferase (ALT) was analyzed by the Clinical Core Laboratories at the Hospital Clinic i Provincial de Barcelona on an ADVIA 2400 Chemistry System (Siemens Medical Solutions, Erlangen, Germany) using commercially available kits, ADVIA Chemistry System 1650 (Bayer-Siemens).

### Hematoxylin and Eosin and Sirius Red Staining

Fresh liver tissue was fixed with 4% paraformaldehyde and embedded in paraffin. The hematoxylin and eosin and Sirius red stainings were performed using standard protocols for the qualitative assessment of liver injury and fibrosis, respectively. The slides were mounted and visualized to evaluate the hepatic histological features.

### Assessment of Oxidized Protein Detection

Detection of oxidized protein in the liver was performed with the OxyIHC oxidized protein detection kit (MilliporeSigma S7450, Burlington, MA, USA) according to the manufacturer’s instructions. Deparaffinized and rehydrated liver sections were covered with antigen retrieval buffer and incubated in a steamer for 20 min. After incubation with 2,4-dinitrophenylhydrazine (DNPH) solution for 30 min at room temperature, sections were incubated with a primary antibody solution, followed by incubation with a biotinylated secondary antibody for 30 min at room temperature. After incubation with streptavidin-conjugated HRP, sections were stained with a DAB (3,3′-diaminobenzidine)-A/B mixture.

### Tissue Immunohistochemistry

Immunohistochemical staining was performed in 7 μm formalin-fixed paraffin-embedded liver sections. The sections were deparaffinized in xylene and dehydrated in a graded alcohol series. Endogenous peroxidase (3% H_2_O_2_), avidin, and biotin were used to block unspecific signal. Slides were incubated with primary antibody (Anti-8-OHdG, sc-66036, Santa Cruz Biotechnology; Anti-F4/80, MCA497G, Bio-Rad; Anti-NLRP3, ab283819, Abcam) overnight in a wet chamber at 4 °C. After washing with PBS, slides were incubated with a biotinylated antibody for 45 min in a wet chamber and developed with an ABC Kit with peroxidase substrate (diaminobenzidine) and peroxidase buffer. After the slides were rinsed with tap water, they were counterstained with hematoxylin and mounted with Aquatex. Olympus BX41 microscope was used to take images. A threshold for percentage of positive area per field was run automatically using Fiji software.

### Statistical Analyses

Statistical analyses were performed using GraphPad Prism 9 (Graphpad Software Inc.). Unpaired Student’s *t*-test (two-tailed) was performed between two groups, and one or two-way ANOVA followed by Tukey’s Multiple Comparison test were used for statistical comparisons between three or more groups. The corresponding number of experiments is indicated in the Figure Legends. Data in graphs are shown as mean ± standard error of the mean (SEM).

## Results

### MCD-Fed Mice Exhibit Hepatocellular Damage, Liver Fibrosis, and Reduced GSH Levels

Despite methionine deprivation-mediated weight loss, the MCD diet is a commonly used model of MASH, characterized by hepatocellular injury, steatosis, inflammation, oxidative stress, and fibrosis.^27,28^ Therefore, we fed mice an MCD diet for up to 15 days (Figure 2A). Consistent with previous reports, serum transaminase levels indicated progressive hepatocellular injury, with ALT levels increasing from day 7 to day 15 of MCD feeding (Figure 2B). Histological analysis using hematoxylin and eosin (H&E) staining confirmed substantial hepatocellular damage by day 14 (Figure 2C). As expected, MCD feeding also induced liver fibrosis, as demonstrated by Sirius Red staining (Figure 2C). In addition, total and mitochondrial liver GSH levels decreased in mice fed the MCD diet as early as 2 days of MCD feeding (Figure 2D), indicating sustained oxidative stress and that intracellular GSH depletion preceded the onset of ALT release and liver injury.

**Figure 2:**
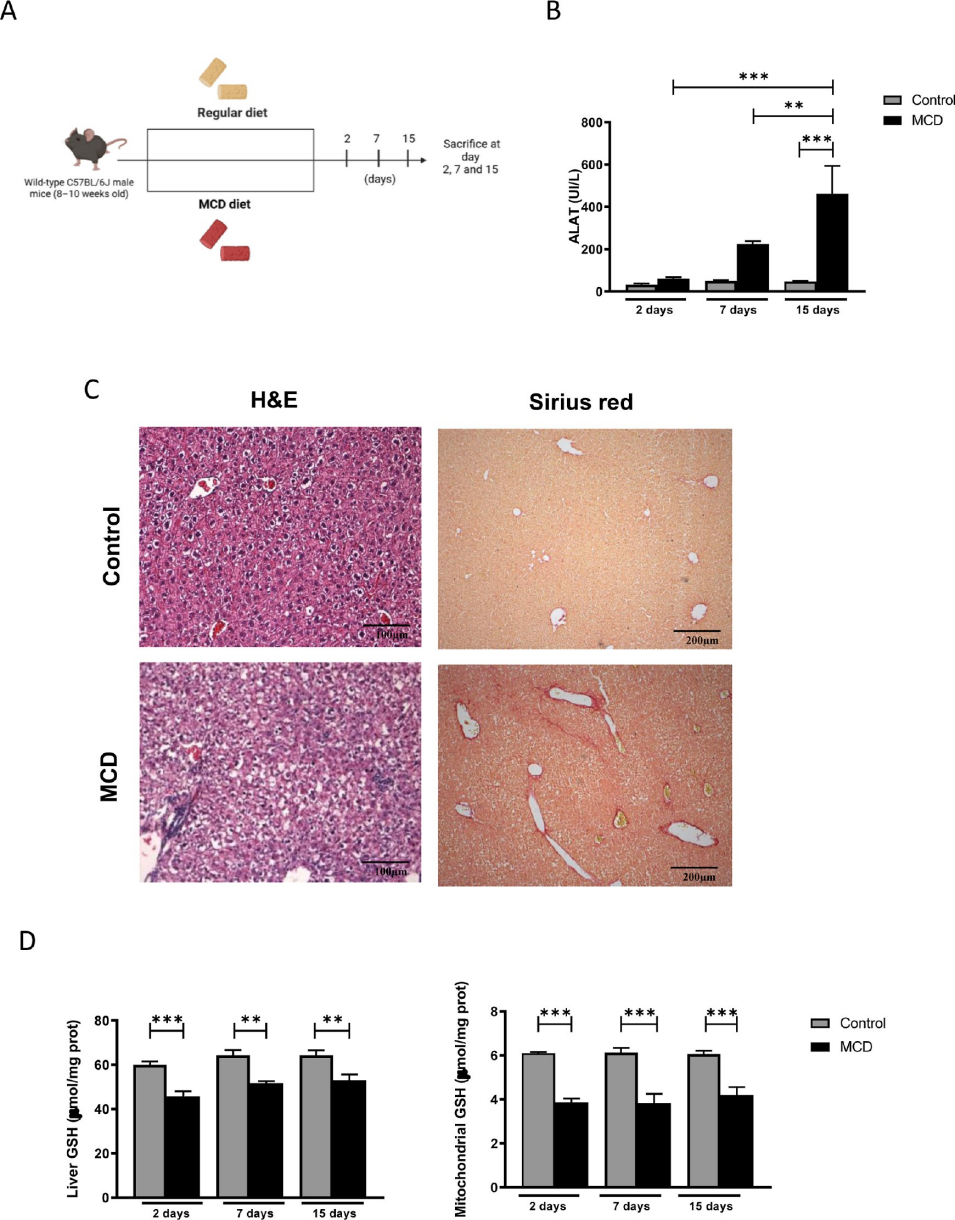
Effect of MCD diet on hepatocellular damage and GSH levels. (A) Experimental design. (B) Serum levels of alanine aminotransferase were measured using standard procedures. (C) Representative hematoxylin and eosin (H&E) and Sirius red staining of liver sections from chow and MCD fed mice at 15 days. Scale bar: 100 or 200 μm. (D) Total and mitochondrial glutathione levels from liver tissue. Data are presented as mean ± SEM (*n* = 4–6 mice per group). **p < 0.01, ***p < 0.001. Statistical significance was determined by one-way ANOVA with Tukey’s post hoc test.

### In Vitro Assessment of l-CySSG in Restoring Mitochondrial Antioxidant Capacity in MCD and HC Dietary Models

To evaluate the capacity of l-CySSG in restoring cytosolic and mitochondrial GSH levels, hepatocytes were isolated from mice fed either the MCD or chow diet for 2 days, time at which mGSH levels are depleted (Figure 2D). This approach with l-CySSG (10 mM) was also performed using NAC and SAM for comparison purposes, to determine the time-dependent replenishment of GSH levels in both compartments (Figure 3A).

**Figure 3:**
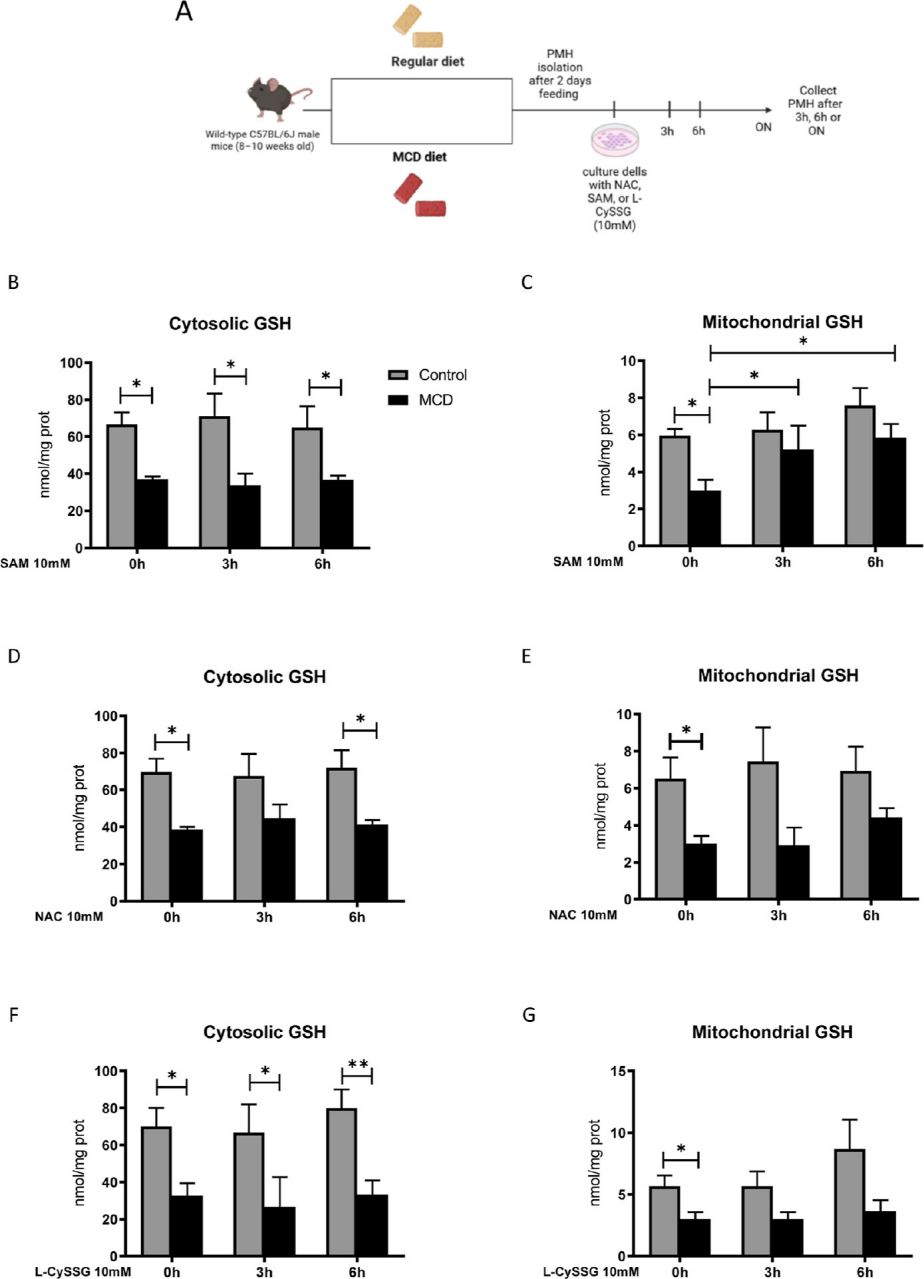
Effectiveness of SAM, NAC, and l-CySSG in modulating GSH levels in hepatocytes. (A) Experimental design of the in vitro treatments. (B) Cytosolic and (C) mitochondrial GSH levels after SAM treatment. (D) Cytosolic and (E) mitochondrial GSH levels after NAC treatment. (F) Cytosolic and (G) mitochondrial GSH levels after l-CySSG treatment. Data represent the mean ± standard deviation of 3 independent experiments. *p < 0.05, **p < 0.01 *t*-test MCD versus control cells. ^#^ *p* < 0.05, ^###^ *p* < 0.001 *t*-test between the indicated groups.

The initial observation revealed a decrease in GSH levels in hepatocytes from mice on MCD diet, regardless of the prodrug’s addition. No changes in cytosolic GSH were observed following treatment at different time points (Figure 3B,D,F). Notably, only SAM effectively restored mGSH levels in MCD hepatocytes up to 3 h, while NAC and l-CySSG did not afford this effect in a significant fashion (Figure 3C,E,G). These results support our previous findings, which suggest that the MCD diet depletes GSH levels and that NAC can only restore mGSH when mitochondrial membrane fluidity is intact.^21,29^

Furthermore, we conducted in vitro experiments using primary hepatocytes exposed to cholesterol along with an ACAT inhibitor, which together mimic cholesterol overload and mitochondrial dysfunction (Supporting Information Figure S1A). In this paradigm, we assessed mitochondrial ROS using MitoSOX and expression of inflammatory markers. Our results demonstrate that cholesterol overload increases mitochondrial ROS production and inflammatory marker expression. Treatment with NAC, l-CySSG, or SAM showed a significant reduction in MitoSOX fluorescence (Figure 1B). In contrast, antioxidant effects on inflammatory markers showed only a trend toward reduction, with significant decreases observed for NAC and SAM specifically in nucleotide-binding oligomerization domain-like receptor protein 3 (Nlrp3) expression (Supporting Information Figure S1C). These effects may occur through distinct mechanisms: SAM by restoring mitochondrial GSH levels and NAC or l-CySSG possibly via direct scavenging of ROS or modulation of signaling pathways involved in inflammation and oxidative stress.

### In Vivo Effect of Different GSH Precursors in a Hypercholesterolemic Animal Model

Changes in PC/PE ratio are responsible of the decrease on membrane fluidity in mice fed MCD diet, and feeding mice SAM replenished mGSH in MCD-fed mice.^21,22^ However, following the hypercholesterolemic dietary model, mitochondrial membrane fluidity is disrupted by increased mitochondrial cholesterol loading.^20^ Consequently, to assess whether the inability of NAC and l-CySSG to restore mGSH was specific for the model of MCD feeding, we analyzed GSH levels following the administration of NAC or l-CySSG in mice subjected to the HC diet for 2 days.

Mice were fed the HC diet for 2 days, followed by treatment with SAM, NAC, or l-CySSG (Figure 4A). Subsequently, we isolated the mitochondria from the livers of these mice to assess GSH levels and we used liver to assess liver injury by H&E analyses. We also measured serum transaminases to assess liver damage associated with the HC diet (Figure 4B). As expected, serum transaminase levels were elevated in the HC mice.^20^ SAM, l-CySSG, and NAC treatment. Therefore, in a clinical context, SAM, NAC, or l-CySSG would provide substantial protection against liver injury caused by the HC diet. Consistent with ALT levels, H&E staining of the liver tissues revealed a significant dilation of liver perisinusoidal spaces after 2 days of HC feeding (Figure 4C), indicating rapid onset of structural liver damage. Mice treated with SAM exhibited signs of recovery, along with a relative improvement observed in those treated with NAC and l-CySSG. Next, we assessed oxidative stress in liver tissue from all groups using the OxyIHC Oxidative Stress Detection Kit, 8-hydroxyguanosine (8-OHG), an indicator of oxidative DNA damage,^30^ and 4-hydroxynonenal (4-HNE), a well-established marker of lipid peroxidation predominantly reflecting mitochondrial oxidative injury.^31^ HC-fed mice had higher baseline oxidation levels compared to controls, and treatment with SAM, NAC, or l-CySSG reduced overall oxidative stress (OxyIHC and 8-OHG; Figure 5A,B, Figure 6A). When specifically analyzing 4-HNE, only SAM significantly reduced its levels, while NAC and l-CySSG did not affect mitochondrial lipid peroxidation (Figure 2A). Regarding total liver GSH levels, we observed that SAM, NAC, and l-CySSG increased the GSH content in the HC-fed mice, which is in line with their roles as GSH precursors in the cytosol (Figure 5C). Moreover, as expected, the HC diet caused a significant depletion of mGSH, which is essential for mitochondrial function and protection against oxidative damage in this organelle, and neither NAC nor l-CySSG was able to restore mGSH within the mitochondria (Figure 5D). In contrast, SAM effectively replenished the mGSH pool, consistent with our previous findings.^17,21^ This capacity to restore mGSH levels makes SAM a particularly promising therapeutic candidate for conditions that involve mitochondrial dysfunction and oxidative stress caused by mitochondrial cholesterol accumulation. Next, we evaluated the expression of the mitochondrial GSH transporter, OGC (2-oxoglutarate carrier), by immunohistochemistry. Our results show that the level of expression of the OGC was significantly reduced in HC mice. Treatment with SAM, NAC, and l-CySSG significantly increased OGC expression levels (Supporting Information Figure S3A). These findings suggest that the protection of HC-fed mice afforded by NAC and l-CySSG may be independent of mGSH restoration. To assess inflammation, we measured F4/80 (for macrophage infiltration) and NLRP3 (an inflammatory marker). Our results show that both markers were significantly increased in HC-fed mice and that treatment with NAC, l-CySSG, or SAM markedly reduced their expression (Figure 6B, Supporting Information Figure S4A). These data support the conclusion that the tested GSH precursors modulate both oxidative stress and inflammation.

**Figure 4:**
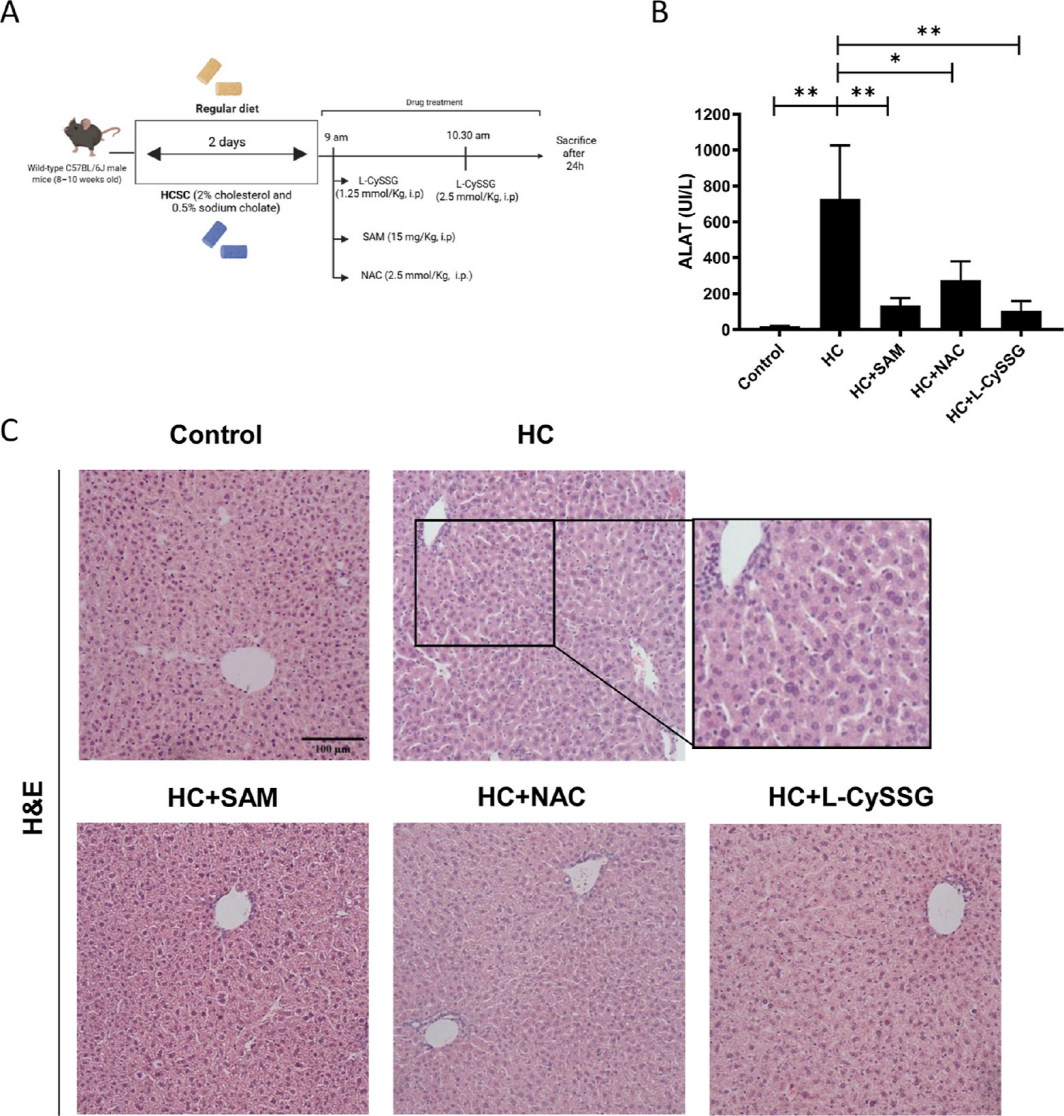
Effects of NAC, l-CySSG, and SAM treatment on liver injury in HC-fed mice. (A) Experimental design. (B) Serum levels of alanine aminotransferase were measured using standard procedures. Data are presented as mean ± SEM (*n* = 4–6 mice per group) *p < 0.05, **p < 0.01. Statistical significance was determined by one-way ANOVA with Tukey’s post hoc test. (C) Representative hematoxylin and eosin (H&E) of liver sections from chow and HC treated and nontreated mice. Scale bar: 100 μm.

**Figure 5:**
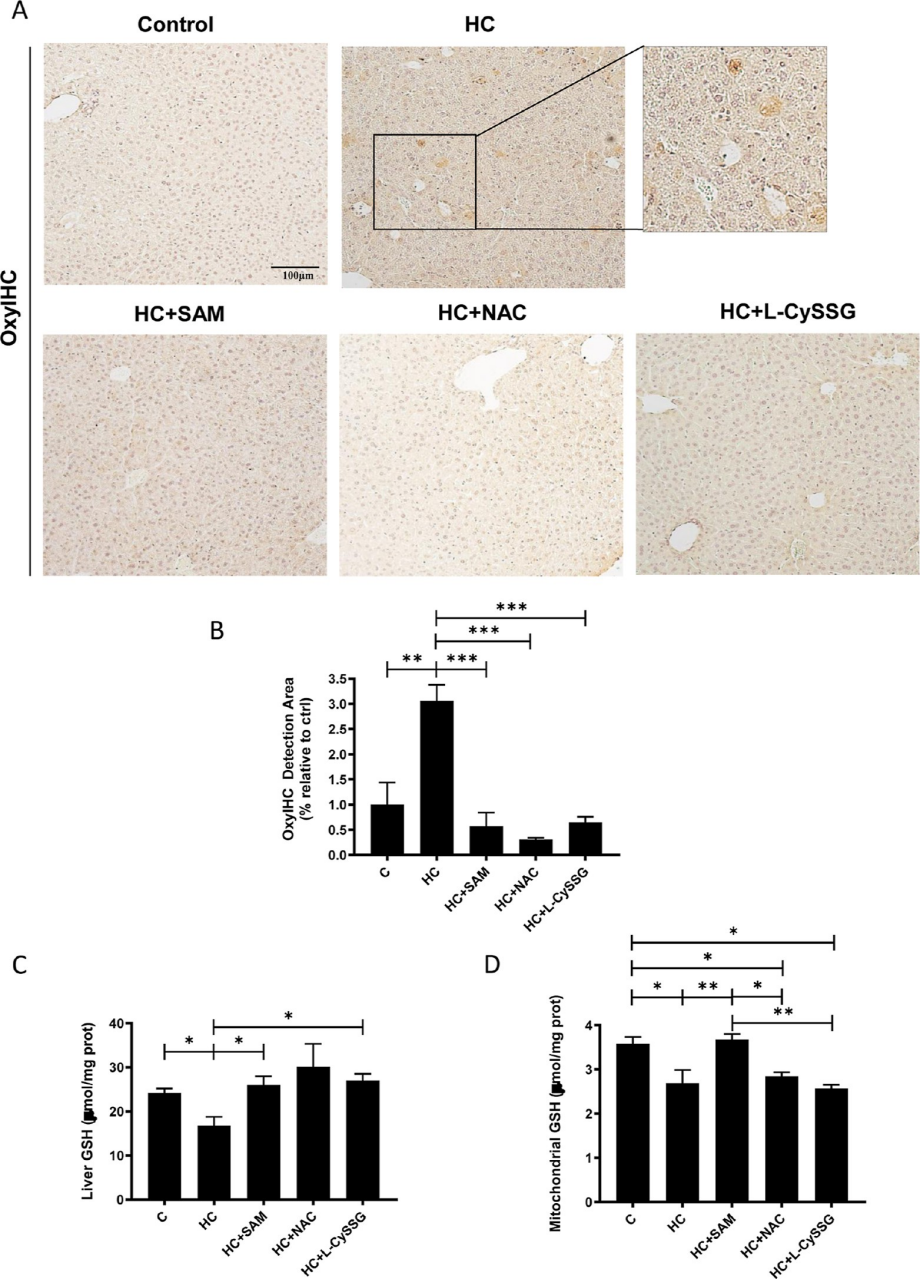
Effects of NAC, l-CySSG, and SAM treatments on protein oxidation levels in HC-fed mice. (A) IHC staining for oxidized proteins of liver sections from chow and HC treated and nontreated mice and (B) quantification. Scale bar: 100 μm. (C) and (D) Total and mitochondrial glutathione levels from liver tissue were measured by Ellman’s assay 21 h after SAM (15 mg/kg), NAC (2.5 mmol/kg) or l-CySSG (2.5 mmol/kg) treatment. Data are presented as mean ± SEM (*n* = 4–6 mice per group). *p < 0.05, **p < 0.01. Statistical significance was determined by one-way ANOVA with Tukey’s post hoc test.

**Figure 6:**
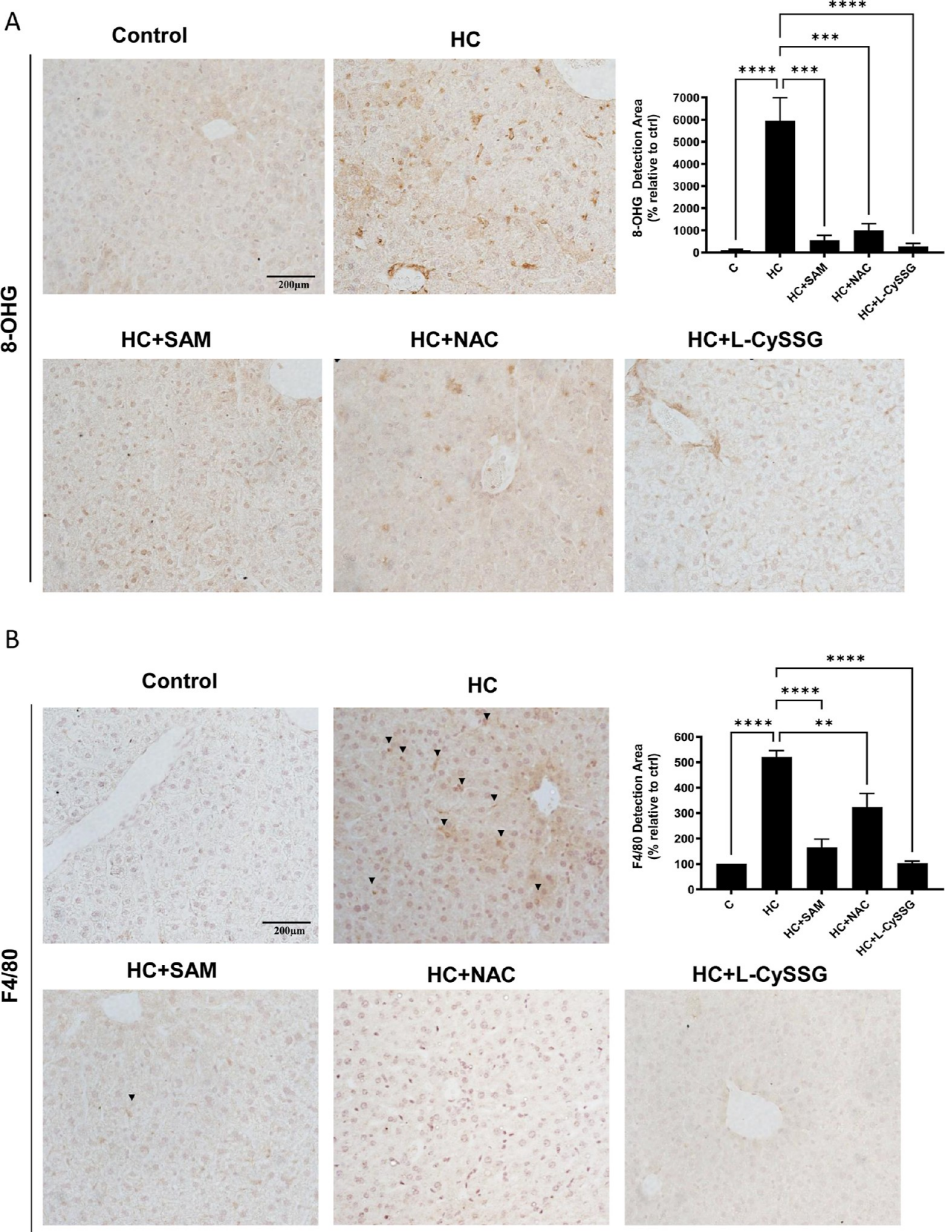
Antioxidant interventions (NAC, l-CySSG, SAM) attenuate oxidative damage and inflammation in Hypercholesterolemic Mice. (A) IHC staining for oxidative DNA (8-hydroxyguanosine, 8-OHG) of liver sections from chow and HC treated and nontreated mice and quantification. (B) IHC staining for macrophage infiltration marker (F4/80) of liver sections and quantification. Scale bar: 200 μm. Data are presented as mean ± SEM (*n* = 4–6 mice per group). *p < 0.05, **p < 0.01. Statistical significance was determined by one-way ANOVA with Tukey’s post hoc test.

### Impact of GSH Precursors on LPS-Induced Liver Injury

LPS are components located within the membranes of bacteria that can cause fulminant hepatitis. This condition arises from a significant release of TNF-α by macrophages in the liver, leading to an excessive generation of ROS. This cascade results in oxidative stress and ultimately triggers apoptosis through the release of cytochrome c from the mitochondria.^32,33^ In this context, HC mice, characterized by a reduced mGSH pool, exhibit heightened sensitivity to LPS.^17^ Consequently, to further investigate the protective effects of SAM, NAC, and l-CySSG against liver damage, we administered LPS to both chow- and HC-diet-fed mice (Figure 7A), subsequently collecting liver tissue and blood samples to assess the extent of injury. Serum analyses indicated that LPS-treated HC mice exhibited transaminase levels that were at least three times higher than those observed in LPS-treated control mice, which were comparable to those in the LPS-untreated control group (Figure 7B). Notably, SAM and l-CySSG, but not NAC, resulted in a reduction of transaminase levels; these results were accompanied by H&E staining (Figure 7B). This finding implies that SAM may play a more effective protective role by replenishing the mGSH pool, as previously reported by our research group.^17,34^ Consistent with the HC model, HC-fed mice challenged with LPS showed elevated protein oxidation relative to chow-fed controls, and oxidative stress markers were reduced following treatment with SAM, NAC, or l-CySSG (Figure 8A,B). In particular, SAM and NAC significantly lowered 4-HNE levels, a marker of lipid peroxidation (Figure 2B). Assessment of OGC expression showed significant recovery following SAM and l-CySSG treatment in HC-LPS mice, while NAC produced a nonsignificant recovery trend (Supporting Information Figure S3B). Finally, analysis of inflammatory markers revealed substantial increases in F4/80 and NLRP3 expression in HC-LPS mice, with all three GSH precursors—NAC, l-CySSG, and SAM—markedly reducing these levels (Figure 9; Supporting Information Figure S4B).

**Figure 7:**
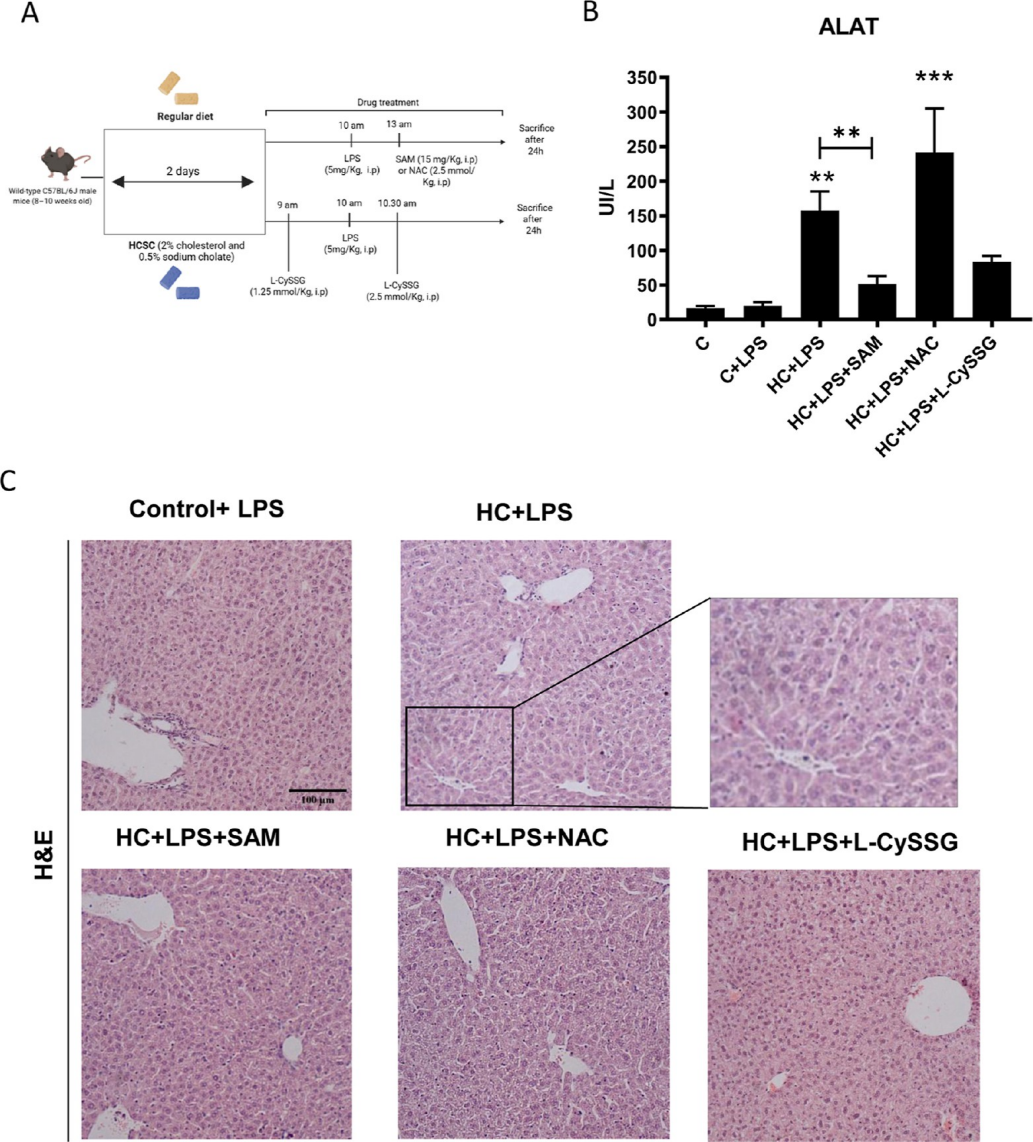
Protection against hepatotoxicity induced by LPS and HC diet through the use of NAC, l-CySSG, and SAM therapies. (A) Experimental design. (B) Serum alanine aminotransferase levels were measured 24 h after LPS (5 mg/kg) administration using standard procedures. Data are presented as mean ± SEM (*n* = 4–6 mice per group). **p < 0.01, ***p < 0.001. Statistical significance was determined by one-way ANOVA with Tukey’s post hoc test. (C) Representative hematoxylin and eosin (H&E) of liver sections. Scale bar: 100 μm.

**Figure 8:**
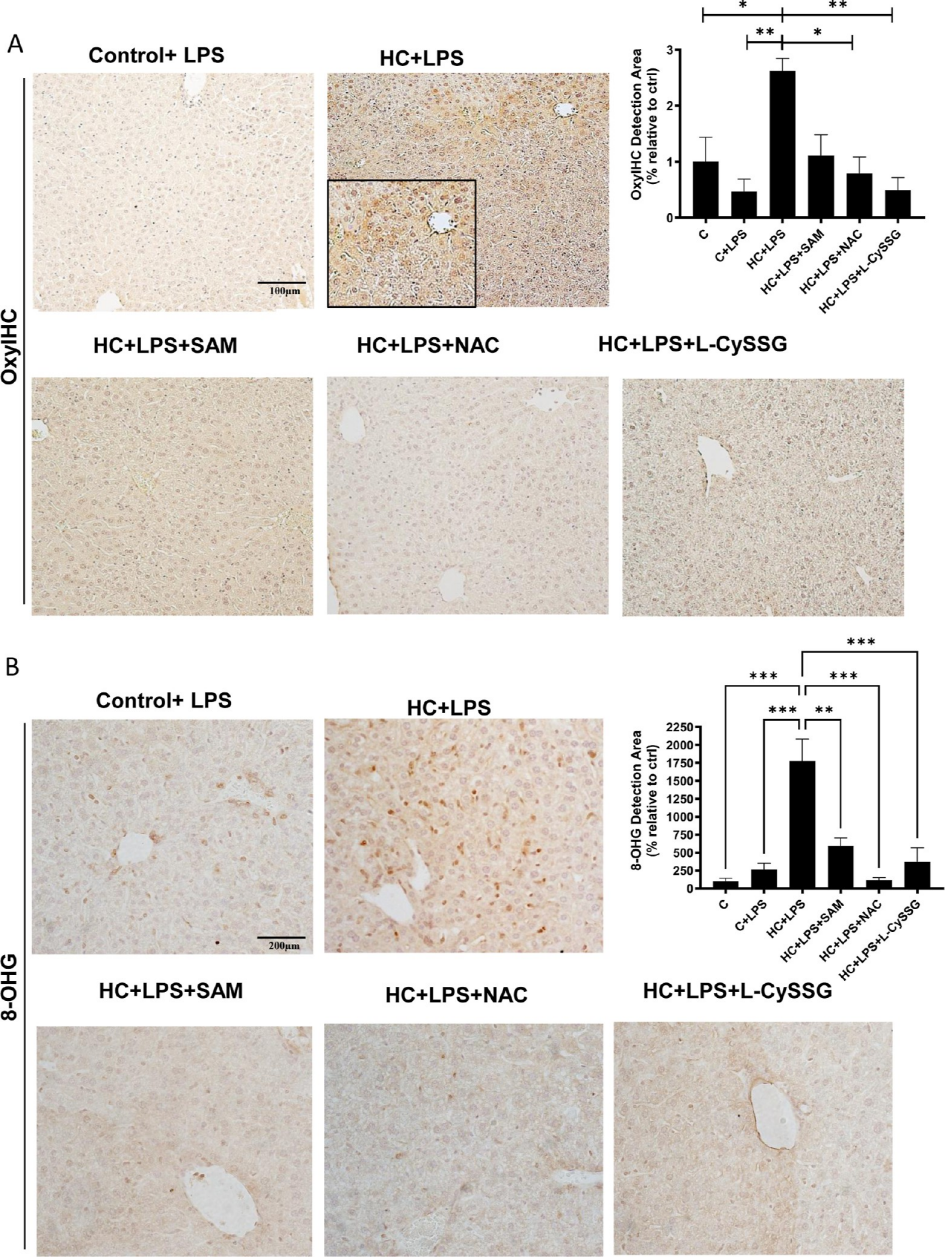
Effect of NAC, l-CySSG, and SAM in protecting against oxidative damage caused by LPS and HC diet. (A) Representative images of the OxyIHC staining of liver sections and quantification. Scale bar: 100 μm. (B) IHC staining for oxidative DNA (8-hydroxyguanosine, 8-OHG) of liver sections from chow and HC treated and nontreated mice and quantification. Scale bar: 200 μm. Data are presented as mean ± SEM (*n* = 4–6 mice per group). *p < 0.05, **p < 0.01. Statistical significance was determined by one-way ANOVA with Tukey’s post hoc test.

**Figure 9:**
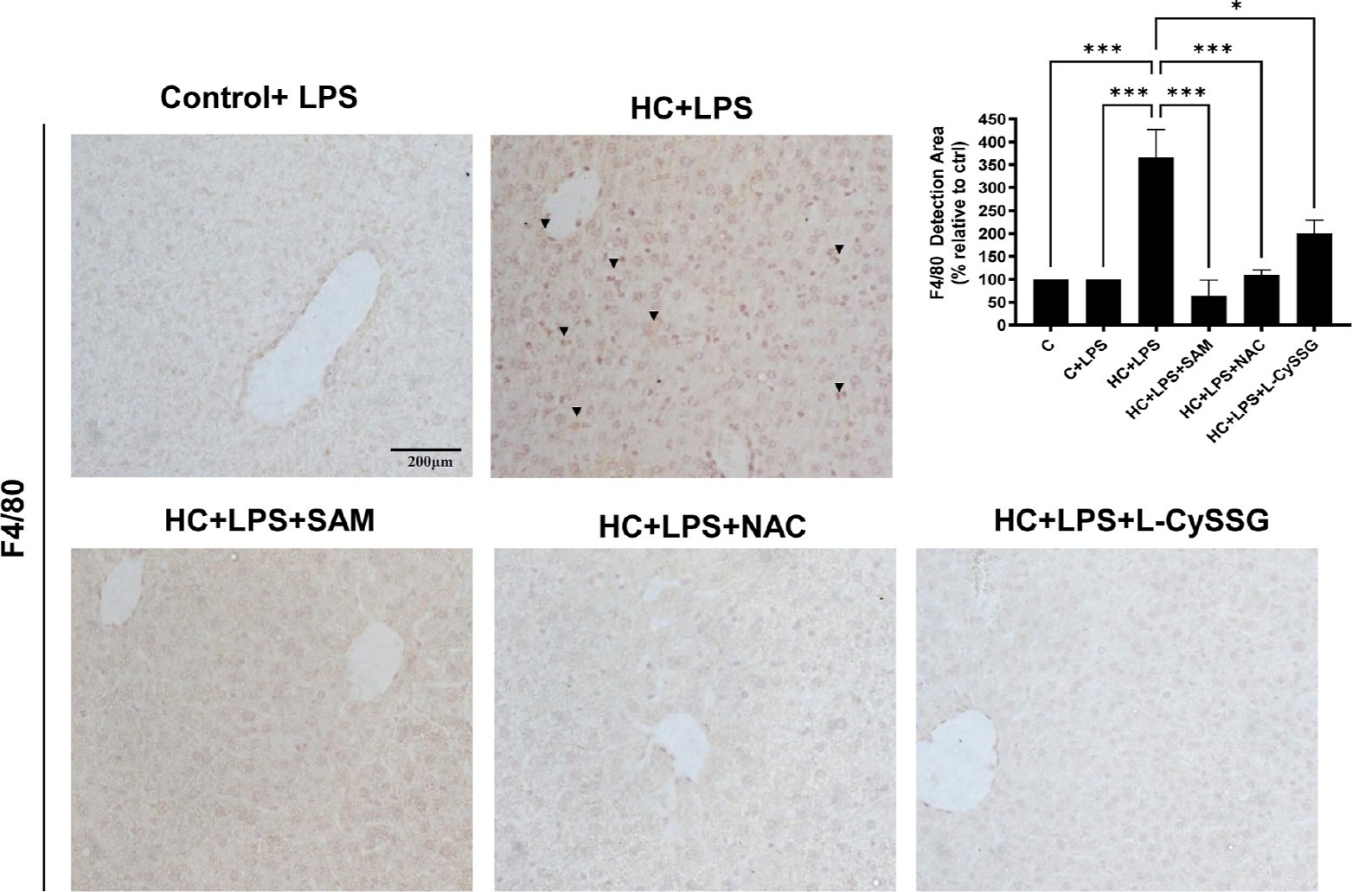
Macrophage Marker F4/80 in Hypercholesterolemic Mice with LPS Induction: Effects of NAC, l-CySSG, and SAM. (A) IHC staining for macrophage infiltration marker (F4/80) of liver sections and quantification. Scale bar: 200 μm. Data are presented as mean ± SEM (*n* = 4–6 mice per group). *p < 0.05, **p < 0.01. Statistical significance was determined by one-way ANOVA with Tukey’s post hoc test.

## Discussion

In this study, we evaluated the hepatoprotective effects of the chemically synthesized compound l-CySSG, along with its ability to affect GSH homeostasis and compartmentalization in cytosol and mitochondria in dietary animal models exhibiting steatohepatitis and hypercholesterolemia. For comparison, we utilized two well-established precursors of GSH, NAC and SAM, which are compounds recognized for their capacity to restore total cellular GSH levels. NAC functions as a precursor of GSH by providing cysteine as the limiting amino acid in the synthesis of GSH catalyzed by GCL.^35^ Additionally, SAM has been shown to serve as a GSH precursor through its conversion into cysteine in the transsulfuration pathway. Furthermore, SAM influences membrane dynamics by altering the PC/PE ratio.^22,36,37^ In contrast, the mechanisms and efficacy of l-CySSG in metabolic liver disease remain less understood.

Previous findings have shown that chronic ethanol exposure, hypercholesterolemia, and steatohepatitis lead to the depletion of mGSH and that SAM, unlike NAC, has the ability to restore mGSH levels when the mitochondrial import of GSH is impaired.^22,25,38–40^ This finding is consistent with the inability of NAC to protect the liver from LPS-induced damage.

Methionine is metabolized to form SAM through the action of methionine adenosyltransferases (MAT), particularly MAT1a and MAT2a.^41^ These enzymes are predominantly expressed in the adult liver as well as in various extrahepatic organs, and SAM is a critical molecule involved in a wide range of biological processes, including methylation reactions, which are essential for DNA, RNA, and protein modification. As a key precursor for the synthesis of other important biomolecules, SAM is crucial for maintaining cellular function and health, particularly in organs such as the liver, where it is involved in detoxification, lipid metabolism, and response to oxidative stress.^42^ Recent research has revealed that SAM is important for preserving liver health and metabolic balance during fasting.^43^ In this process, the levels of SAM in the liver decline, which aids the body in adjusting to the absence of food. This decrease is significant because it regulates the relationship between the endoplasmic reticulum and mitochondria, which are both essential for energy production and metabolism. During fasting, MAT1A is activated in specific areas known as mitochondria-associated membranes, which fuel phosphatidylethanolamine *N*-methyltransferase (PEMT) activity, involved in lipid metabolism. Furthermore, the synthesis of SAM during fasting protects the endoplasmic reticulum from stress and potential liver damage. In summary, SAM is essential for maintaining liver health and metabolic balance during fasting rather than simply being a byproduct of metabolism.^43^

The distinguishing factor between SAM and NAC lies in SAM’s ability to facilitate the import of GSH into mitochondria. Consequently, in situations where mitochondrial GSH transport is disrupted through decreased membrane fluidity, NAC fails to elevate mGSH levels, resulting in a predisposition to oxidative stress and diminished protective effects against injury.^44^ Additionally, prior work demonstrated that GSH ethyl ester (GSH-EE), a membrane-permeable GSH derivative, effectively restores mGSH levels in models where mitochondrial GSH transport is defective, including Niemann-Pick type C (NPC) disease and dietary/genetic MASH models. For example, in NPC disease, a lysosomal disorder, NAC facilitates GSH synthesis in the cytosol by providing cysteine but does not restore mGSH levels in Npc1^–/–^ mice due to the block of mitochondrial GSH transport imposed by increased cholesterol accumulation. However, administration of GSH-EE to Npc1^–/–^ mice restored mGSH levels, indicating its ability to diffuse to mitochondria, bypassing the restricted effect of cholesterol-mediated decrease in membrane fluidity. This restoration led to improved mitochondrial function, reduced oxidative stress, reduced liver injury, reversed locomotor deficits, and a longer lifespan for the mice.^29^ Similarly, in nutritional and genetic MASH models using MAT1A knockout mice and MCD feeding, GSH-EE restores mGSH levels, while NAC fails to recover the mGSH pool and to protect both models against oxidative stress that was worsened by superoxide anion scavenging.^45^ Thus, GSH-EE has a unique ability to restore mGSH levels by directly supplying GSH into mitochondria, bypassing the active transport of GSH through specific carriers whose activity is sensitive to mitochondrial membrane dynamics.

The findings regarding l-CySSG highlight its potential as a protective agent in liver health despite its limited ability to restore mGSH levels, as opposed to SAM or GSH-EE. However, the fact that l-CySSG provides protection even without restoring mGSH levels suggests that there might be other mechanisms at play, independent of the restoration of the specific pool of mitochondrial GSH. This raises intriguing questions about how l-CySSG exerts its protective effects, potentially involving cellular pathways or signaling mechanisms not yet fully understood. One possibility is that while both SAM and NAC boost cysteine levels, their impact on GSH content relies on endogenous GSH biosynthesis, and l-CySSG upon hydrolysis may provide GSH directly, bypassing the susceptibility of CGL to oxidative stress-mediated modifications of critical cysteine residues.

Another additional difference between l-CySSG and other GSH-related compounds is its bioavailability. While oral GSH supplementation is often ineffective due to enzymatic degradation by gamma-glutamyl transpeptidase^46,47^ l-CySSG is bioavailable when taken orally in mice. This ability to be absorbed and utilized effectively in the body without the same degradation could make l-CySSG a promising alternative for therapeutic purposes, particularly in protecting against liver damage caused by toxins like acetaminophen.^23^

l-CySSG has been investigated as a protective agent against liver damage caused by xenobiotics, acting as a sulfhydryl-modified prodrug of GSH.^24^ It effectively protects mice from acetaminophen-induced hepatotoxicity, unlike D-CySSG, suggesting that GSH is released from l-CySSG through enzymatic reduction or thiol–disulfide exchange. l-CySSG’s ability to be generated both inside and outside the cell through thiol–disulfide exchange with cystine further adds to its versatility as a GSH prodrug.^48^ This reaction can occur spontaneously or via the enzymatic action of thioltransferases,^49^ and it may be facilitated by gamma-glutamyltransferase (GGT), which can convert GSSG (oxidized GSH) into l-CySSG and cystine. The potential for modulating this reaction, such as by inhibiting GGT with OU749,^50^ could have therapeutic applications in cancer cells, particularly in tumors where GGT activity is upregulated to support the high oxidative stress environment.

Additionally, studies by Phimister et al. demonstrated that l-CySSG can deliver GSH to Clara cells during naphthalene exposure, reducing protein adducts from reactive metabolites and limiting GSH depletion.^51^ Although its mechanism of action is not fully understood, l-CySSG shows potential as a therapeutic tool for enhancing the thiol pool due to its efficacy and stability.

Moreover, the role of circulating l-CySSG and other glutathionergic compounds in calcium regulation is another relevant aspect of its action. By interacting with the calcium-sensing receptor, these compounds can enhance the receptor’s sensitivity to extracellular calcium, thus influencing calcium homeostasis in various organs like the parathyroids, kidneys, and bones.^3^ This mechanism suggests that l-CySSG may have broader physiological effects beyond its antioxidant and hepatoprotective roles, potentially affecting a wide range of cellular processes that depend on calcium regulation.

Importantly, our in vivo findings demonstrate that all three GSH precursors—SAM, NAC, and l-CySSG—modulate oxidative stress, inflammation, and mitochondrial transporter expression, albeit with distinct efficacies. LPS-treated HC-fed mice showed markedly increased oxidative stress, evidenced by elevated protein oxidation and lipid peroxidation markers, which were significantly reduced by SAM and partly by NAC and l-CySSG treatments. Notably, only SAM significantly restored mitochondrial GSH levels, underlying its capacity to counteract the restriction of mitochondrial GSH transport akin to GSH-EE. Moreover, the expression of the mitochondrial GSH transporter, OGC was significantly reduced in HC and LPS-treated HC mice that was rescued by SAM and l-CySSG treatments; NAC showed a trend toward improvement without reaching significance in LPS-treated HC mice. This suggests that the restoration of transporter expression may be key for mitochondrial GSH homeostasis and effective protection against oxidative injury. However, whether the activity of the OGC is sensitive to the oxidative stress environment remains to be further investigated. Inflammatory markers—F4/80 (macrophage infiltration) and NLRP3 (inflammasome component)—were increased in HC-fed and LPS-challenged mice but decreased significantly upon treatment with all three precursors. These results indicate that while SAM’s effects may relate to enhanced mitochondrial antioxidant capacity, l-CySSG and NAC also exert meaningful anti-inflammatory actions and reduce oxidative stress, contributing to hepatoprotection through complementary mechanisms.

Supporting the in vivo results, in vitro experiments employing hepatocytes from mice fed a short-term MCD diet, which depletes mitochondrial GSH, showed that treatment with SAM, NAC, or l-CySSG did not alter the cytosolic GSH levels. Importantly, only SAM significantly restored mitochondrial GSH levels up to 3 h post-treatment, while NAC and l-CySSG had no significant effect under these conditions. This supports the idea that SAM effectively replenishes mitochondrial GSH even when the membrane integrity is compromised. Complementing this, primary hepatocytes exposed to cholesterol overload exhibited increased mitochondrial ROS production and inflammatory marker expression. NAC, l-CySSG, and SAM each significantly reduced mitochondrial ROS, with NAC and SAM notably decreasing Nlrp3 expression. These findings suggest that SAM’s main protective action is through restoring mitochondrial GSH, whereas NAC and l-CySSG primarily act by scavenging ROS or modulating stress-responsive signaling. Together, these results highlight the complementary antioxidant and anti-inflammatory mechanisms by which GSH precursors protect liver cells.

In summary, l-CySSG emerges as a compound with significant therapeutic potential, particularly for liver protection, even though its mechanism of action may differ from other GSH-related compounds like SAM and NAC. Its oral bioavailability, ability to modulate oxidative stress and inflammation, partial restoration of mitochondrial transporter expression, and potential influence on calcium regulation open new avenues for research and clinical application, particularly in diseases involving oxidative stress and mitochondrial dysfunction. Further exploration of its mechanisms and comparative analyses with GSH-EE will be valuable to fully elucidate l-CySSG’s clinical potential.

## Supplementary Material



## Notes

Additional materials and methods, mitoSOX fluorescent levels after cholesterol treatment and SAM, NAC, l-CySSG in cultured hepatocytes, modulation of 4-HNE expression by NAC, l-CySSG, and SAM in hypercholesterolemic mice with or without LPS induction, effects of NAC, l-CySSG, and SAM on OGC transporter expression in hypercholesterolemic mice with or without LPS induction, and inflammasome marker NLRP3 in hypercholesterolemic mice with or without LPS induction (PDF)

## ∇

Deceased during the performance of this study (28th August 2024).

## Abbreviations

4-HNE: 4-hydroxynonenal
8-OHG: 8-hydroxyguanosine
ALT: alanine aminotransferase
CaSR: calcium-sensing receptor
DAB: 3,3′-diaminobenzidine
DNPH: 2,4-dinitrophenylhydrazine
GCL: glutamate-cysteine ligase
GS: glutathione synthetase
GSH: glutathione
GSH-EE: GSH ethyl ester
GSSG: oxidized glutathione
HC: hypercholesterolemic
H&E: hematoxylin and eosin
i.p: intraperitoneally
l-CySSG: l-cysteine-glutathione disulfide
LPS: lipopolysaccharides
MASLD: metabolic dysfunction-associated steatotic liver disease
MAT: methionine adenosyltransferase
MCD: methionine-choline deficient diet
mGSH: mitochondrial GSH
NAC: *N*-acetylcysteine
NLRP3: nucleotide-binding oligomerization domain-like receptor protein 3
NPC: Niemann-Pick type C
OGC: 2-oxoglutarate carrier
ON: overnight
PC: phosphatidylcholine
PE: phosphatidylethanolamine
PEMT: phosphatidylethanolamine *N*-methyltransferase
SAM: *S*-adenosylmethionine
VLDL: very low-density lipoproteins
