# Supplementary material for: l‑Cysteine-Glutathione Mixed Disulfide, a Novel Bioavailable Sulfhydryl-Modified Glutathione Precursor, Protects against Early Liver Injury Induced by Short-Term Hypercholesterolemia
Source: Chem Res Toxicol. 2025 Oct 23;38(11):1961–76. doi: 10.1021/acs.chemrestox.5c00272 (PMC12628329; doi:10.1021/acs.chemrestox.5c00272)

SUPPORTING INFORMATION

L-Cysteine-Glutathione Mixed Disulfide, a novel bioavailable sulfhydryl-modified glutathione precursor, protects against early liver injury induced by short-term hypercholesterolemia.

AUTHOR NAMES

Laura Martínez-Gili[1,2,3#], Raquel Fucho[1,2,3#], Francisco Caballero[1,2,3#], Susana Núñez[1,2,3], Hala Saeed Jaara[1,2,3], Cristina Alarcón-Vila[1,2,3], Naira Rico[4], Herbert T Nagasawa[5†], Carmen García-Ruiz[1,2,3,6], José C Fernández-Checa[1,2,3,6], Sandra Torres[1,2,3*]*

AUTHOR ADDRESS

[1]Department of Molecular and Cellular Biomedicine, Institute of Biomedical Research of Barcelona (IIBB), Spanish National Research Council (CSIC), 08036 Barcelona, Spain.

[2]Liver Unit, Hospital Clinic I Provincial de Barcelona, Institut d'Investigacions Biomèdiques August Pi i Sunyer (IDIBAPS), 08036 Barcelona, Spain.

[3]Center for the Study of Liver and Gastrointestinal Diseases (CIBERehd), Carlos III National Institute of Health, 28029 Madrid, Spain.

[4]CORE Laboratory, Biochemistry and Molecular Genetics Department, Biomedical Diagnostic Centre, Hospital Clínic, Barcelona, Spain.

[5] Center for Drug Design, University of Minnesota, Minneapolis, MN 55455, USA.

[6]Division of Gastrointestinal and Liver Diseases, Department of Medicine, Keck School of Medicine, University of Southern California, Los Angeles, CA 90033, USA.

#These authors contributed equally to the work.

†Deceased during the performance of this study (28th August 2024)

Contact information

*Correspondence: sandra.torres@iibb.csic.es (S.T.); Tel.: +34 93 227 40 21; checa229@yahoo.com or josecarlos.fernandezcheca@iibb.csic.es (J.C.F.-C.T); Tel.: +34 93 227 57 09.

Supporting Information – Table of Contents

Title	Page Number
SUPPLEMENTARY MATERIALS AND METHODS	3
FIGURE LEGENDS	4
SUPPLEMENTARY FIGURE 1	5
SUPPLEMENTARY FIGURE 2	6
SUPPLEMENTARY FIGURE 3	7
SUPPLEMENTARY FIGURE 4	8

SUPPLEMENTARY MATERIALS AND METHODS

Tissue immunohistofluorescence

For immunohistofluorescence analyses, 12-µm liver frozen sections were fixed in 10% formalin for 1 h at room temperature, washed with PBS and blocked with antibody diluent (Abcam ab64211) and 10% Normal Goat Serum ready-to-use (Life technologies #5000627) at room temperature for 20 and 10 min, respectively. Sections were then washed with PBS and incubated overnight at 4°C with the following primary antibodies: Anti-OGC (Santa Cruz, sc-515593), and Anti-4-HNE (Abcam, ab46545). Sections were subsequently washed with PBS, incubated with their respective fluorochrome-conjugated secondary antibodies for 45 min at room temperature. Samples were finally washed with PBS and embedded in ProLong™ Diamond Antifade Mountant with DAPI (Invitrogen, P36962) to mount the coverslips. Digital images were taken in a Leica DM2500 confocal microscope.

Mitochondrial determination of superoxide anion

Hepatocytes from HC were incubated with MitoSOX (5mM, Molecular Probes) for 30 min at 37 °C in the dark, washed with PBS and evaluated by fluorescence spectroscopy using the Tecan Infinite® 200 PRO multi-mode microplate reading (640 nm for excitation and 665 nm for emission). The fluorescence was normalized by protein content, determined by BCA protein assay.

Real time PCR

Total RNA was isolated from mouse brain samples with Trizol reagent (Invitrogen, Carlsbad, CA). Quantitative Reverse Transcription Polymerase Chain Reaction (qRT-PCR) was performed using the SensiFAST™ SYBR® No-ROX 2-step Mix 2x kit (Bioline, BIO-98020) on a Biorad CFX384 system, following the manufacturer's instructions. Each reaction was run in triplicate. Quantification of a given gene, expressed as relative mRNA level compared with control, was calculated after normalization to the housekeeping gene, 18S, and determined using the 2-ΔΔCt method. The following primer sequences synthesized from Invitrogen were used: Il1β forward 5'- GAGCTGAAAGCTCTCCACCTC-3'; Il1β reverse 5'-CTTTCCTTTGAGGCCCAAGGC-3'; Nlpr3 forward 5'-AAGTAAGGCCGGAATTCACC-3'; Nlpr3 reverse 5'-GCTCCAACCATTCTCTGACC-3'; Actin forward 5'-GACGGCCAGGTCATCACTAT-3'; Actin reverse 5'-CGGATGTCAACGTCACACTT-3'.

SUPPLEMENTARY FIGURE LEGENDS

Supplementary Figure 1. MitoSOX fluorescent levels after cholesterol treatment and SAM, NAC and L-CySSG in cultured hepatocytes. A) Experimental design of the *in vitro* treatments. B) MitoSOX fluorescent levels. C) Gene expression of inflammatory markers. Data represent the mean ± standard deviation (SD) of 3 independent experiments. *p < 0.05, **p<0.01, ***p<0.001. Statistical significance was determined by one-way ANOVA with Tukey's post-hoc test.

Supplementary Figure 2. Modulation of 4-HNE Expression by NAC, L-CySSG, and SAM in Hypercholesterolemic Mice with or without LPS Induction. IF staining for 4-HNE of liver sections and quantification A) from chow and HC treated mice or B) LPS-treated HC mice. Scale bar: 25µm. Data are presented as mean ± SEM (n= 4-6 mice per group). *p < 0.05, **p<0.01. Statistical significance was determined by one-way ANOVA with Tukey's post-hoc test.

Supplementary Figure 3. Effects of NAC, L-CySSG, and SAM on OGC Transporter Expression in Hypercholesterolemic Mice with or without LPS Induction. IF staining for OGC of liver sections and quantification A) from chow and HC treated mice or B) LPS-treated HC mice. Scale bar: 25µm. Data are presented as mean ± SEM (n= 4-6 mice per group). *p < 0.05, **p<0.01. Statistical significance was determined by one-way ANOVA with Tukey's post-hoc test.

Supplementary Figure 4. Inflammasome Marker NLRP3 in Hypercholesterolemic Mice with or without LPS Induction: Effects of NAC, L-CySSG, and SAM. IHC staining for NLRP3 of liver sections and quantification from A) chow and HC treated mice or B) LPS-treated HC mice. Scale bar: 200µm. Data are presented as mean ± SEM (n= 4-6 mice per group). *p < 0.05, **p<0.01. Statistical significance was determined by one-way ANOVA with Tukey's post-hoc test.

Supplementary Figure 1. MitoSOX fluorescent levels after cholesterol treatment and SAM, NAC and L-CySSG in cultured hepatocytes

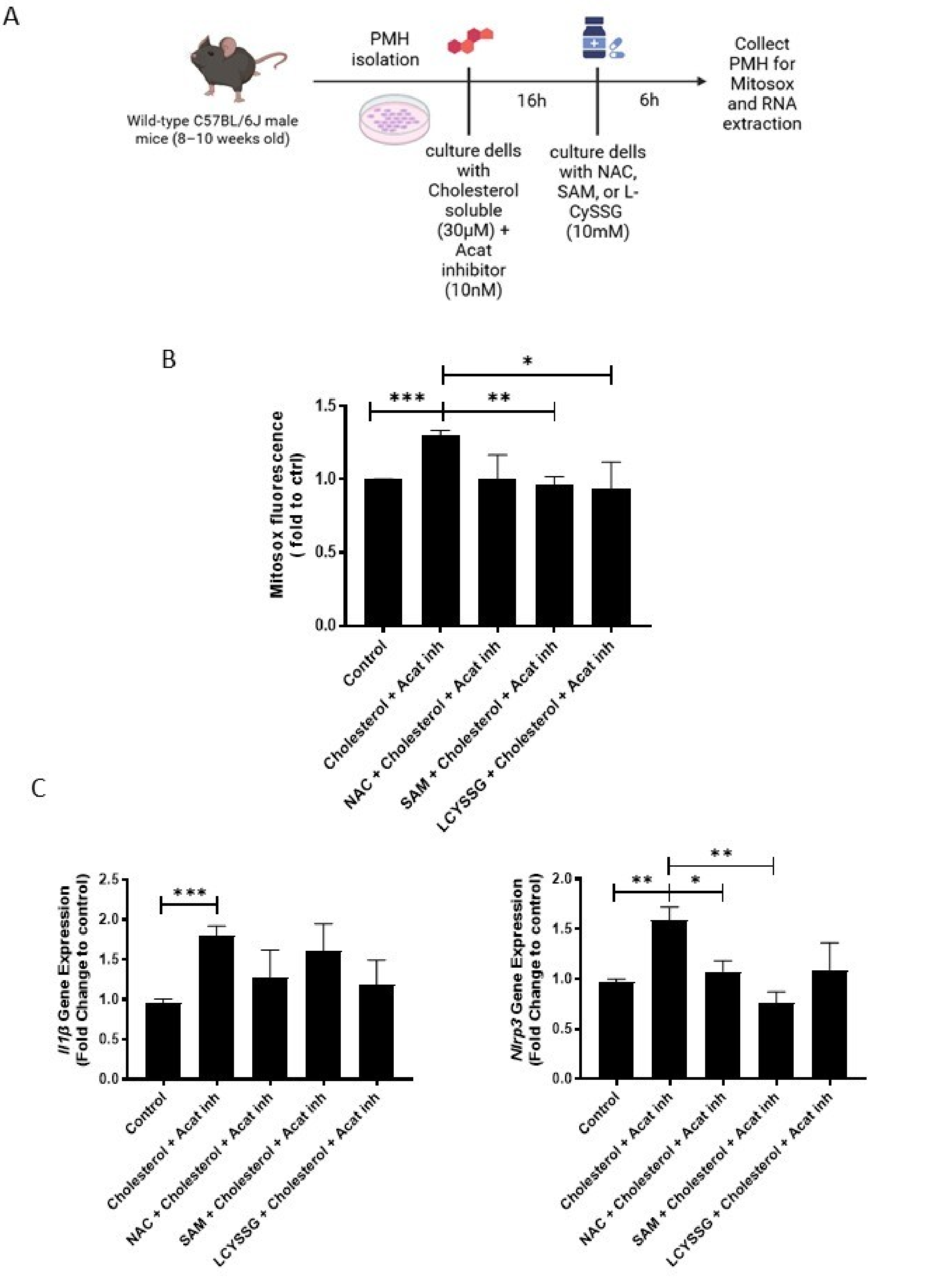

Supplementary Figure 2. Modulation of 4-HNE Expression by NAC, L-CySSG, and SAM in Hypercholesterolemic Mice with or without LPS Induction

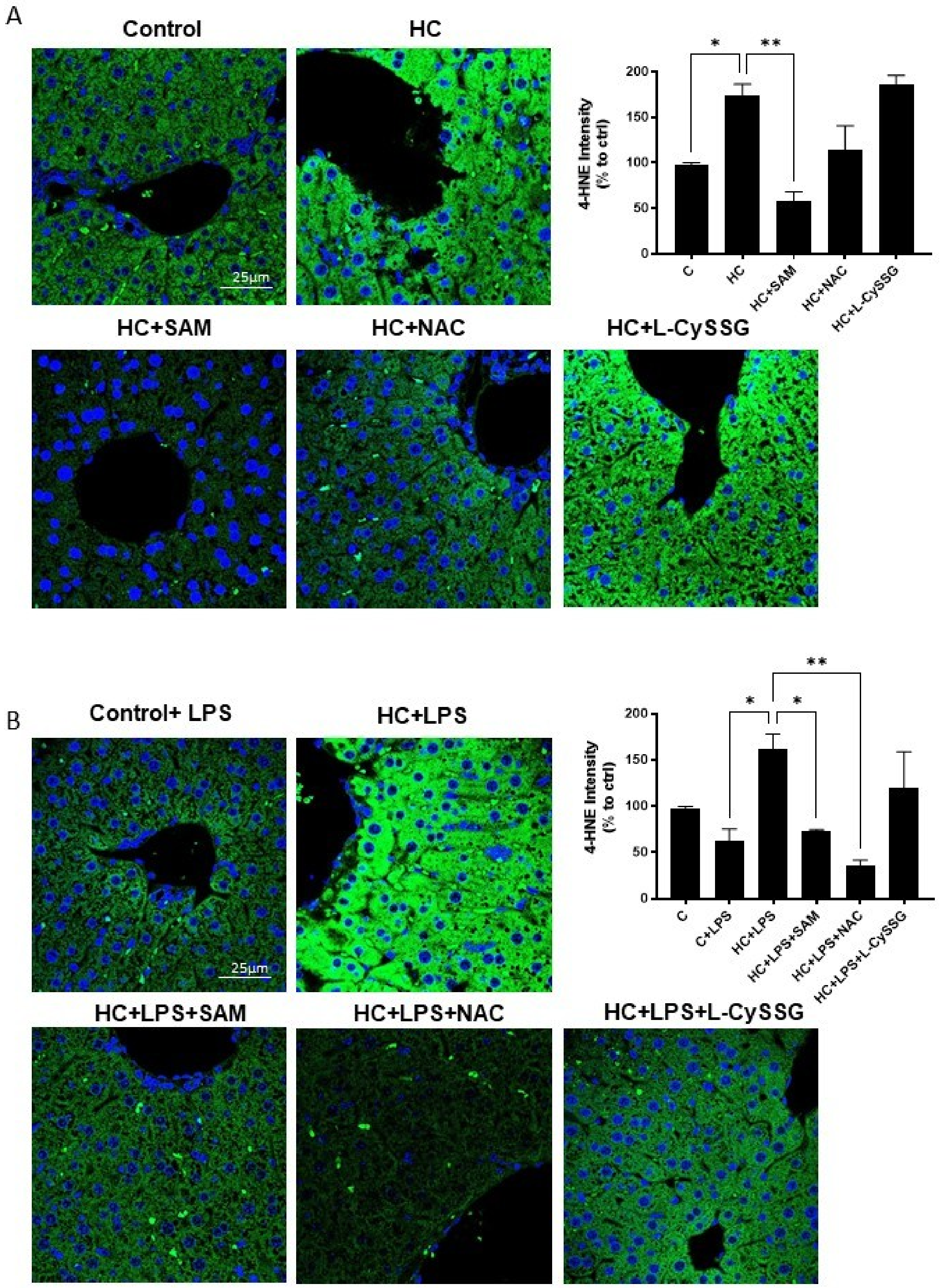

Supplementary Figure 3. Effects of NAC, L-CySSG, and SAM on OGC Transporter Expression in Hypercholesterolemic Mice with or without LPS Induction

A

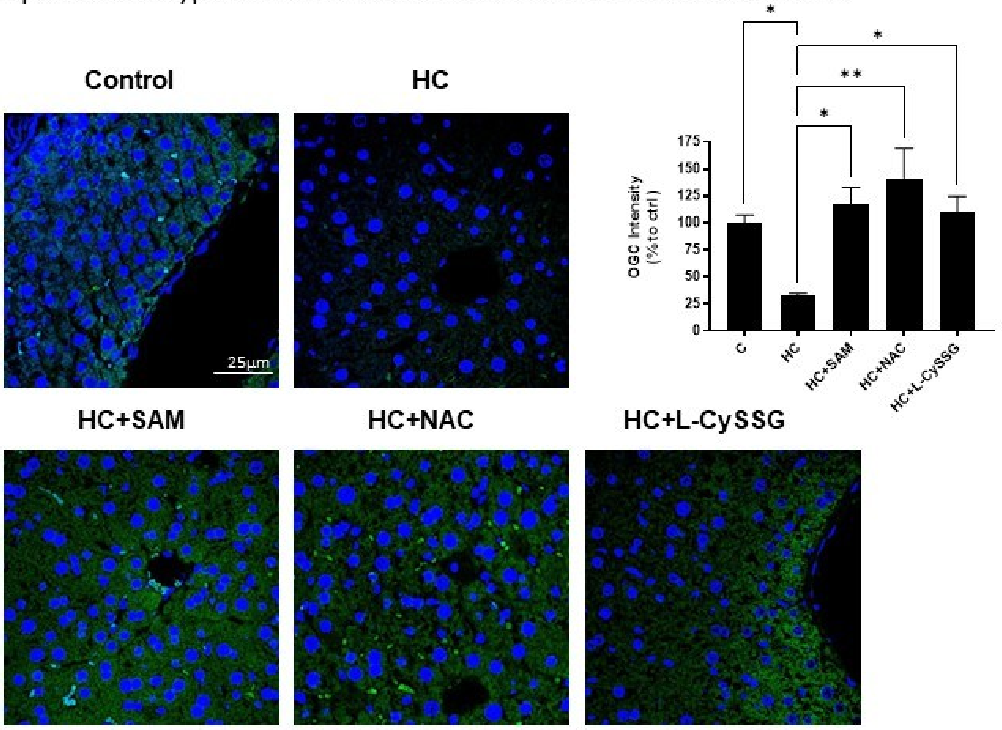

B

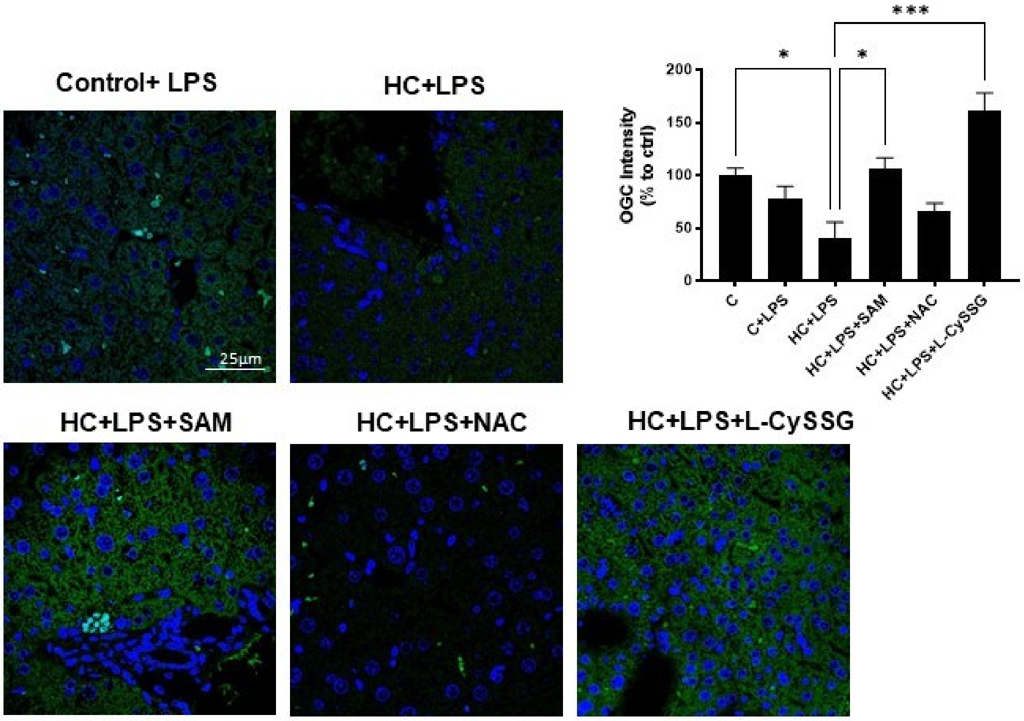

Supplementary Figure 4. Inflammasome Marker NLRP3 in Hypercholesterolemic Mice with or without LPS Induction: Effects of NAC, L-CySSG, and SAM

A

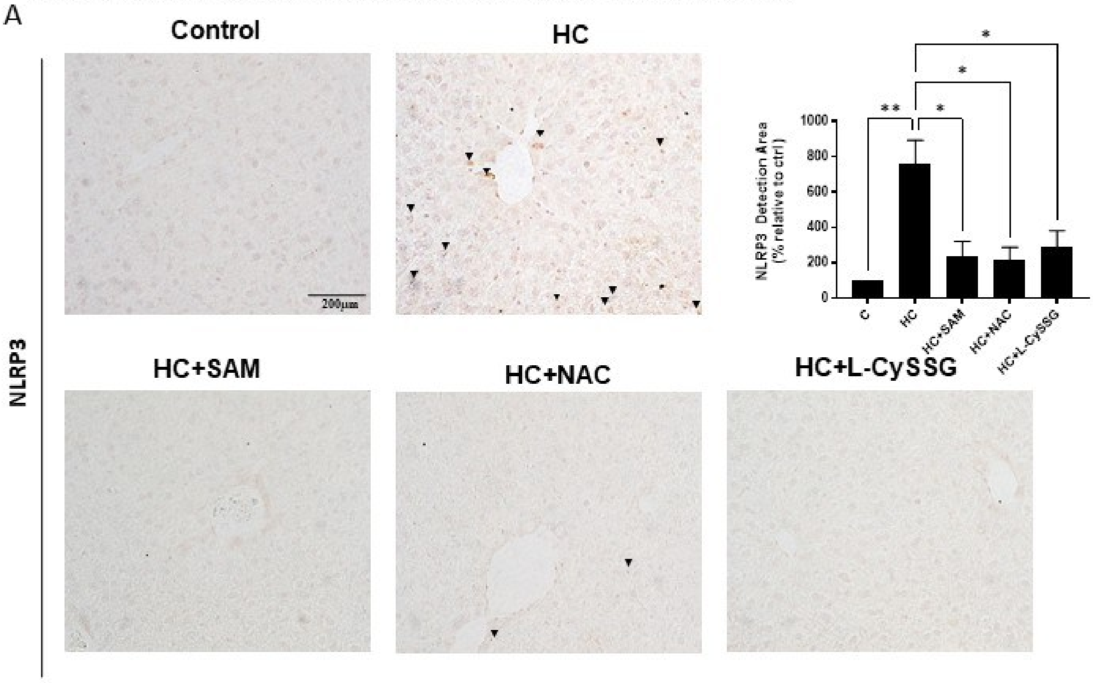

B

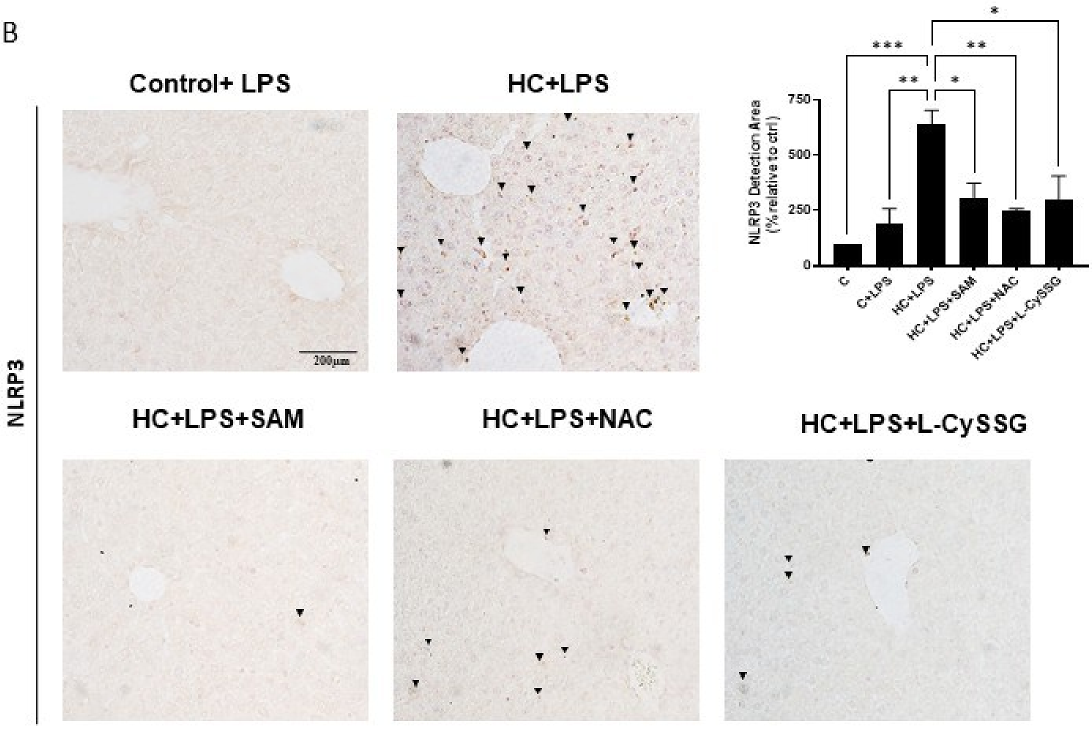

Supplement: Supplementary file 1 [file tx5c00272_si_001.pdf]
